# Hypertension identified in early adulthood as an endocrine–vascular risk factor for uterine fibroids in reproductive-age women: a nationwide cohort study

**DOI:** 10.3389/fendo.2026.1822452

**Published:** 2026-08-25

**Authors:** Soyeon Kang, Kyungdo Han, Youn-Jee Chung

**Affiliations:** 1 Division of Reproductive Endocrinology, Department of Obstetrics and Gynecology, St. Vincent’s Hospital, College of Medicine, The Catholic University of Korea, Seoul, Republic of Korea; 2 Department of Statistics and Actuarial Science, Soongsil University, Seoul, Republic of Korea; 3 Division of Reproductive Endocrinology, Department of Obstetrics and Gynecology, Seoul St. Mary’s Hospital, College of Medicine, The Catholic University of Korea, Seoul, Republic of Korea

**Keywords:** blood pressure, early-adulthood hypertension, endocrine–vascular dysregulation, reproductive-age women, uterine fibroid

## Abstract

**Introduction:**

The association between hypertension identified in early adulthood and uterine fibroid development in young women remains poorly understood. Therefore, we aimed to evaluate the association between hypertension identified in early adulthood and uterine fibroid development in reproductive-age women.

**Methods:**

In this nationwide population-based cohort study, we used data from the Korean National Health Insurance Service, health screening and claims database. A total of 2,558,659 women aged 20–39 years without a prior diagnosis of uterine fibroids were enrolled between 2009–2012 and followed for a mean duration of 11.54 years until December 31, 2023. The main outcome measures were incident uterine fibroids and adjusted hazard ratios (HRs) according to blood pressure categories. Blood pressure was categorized as normotension, elevated/stage 1 hypertension, stage 2 hypertension, or treated hypertension according to national guidelines. Incident uterine fibroids were identified using International Classification of Disease 10^th^ revision code D25. Cox proportional hazards models were used to estimate HRs and 95% confidence intervals (CIs), adjusting for demographic factors, lifestyle behaviors, body mass index (BMI), metabolic comorbidities, and healthcare utilization.

**Results:**

During follow-up, 373,379 women (14.59%) developed uterine fibroids. Compared with normotensive women, elevated/stage 1 hypertension (adjusted HR 1.04; 95% CI 1.03–1.05) and stage 2 hypertension (adjusted HR 1.06; 95% CI 1.04–1.09) were associated with a progressively increased risk of incident uterine fibroids (all *P* < 0.001). Women receiving antihypertensive medication exhibited the highest risk (adjusted HR 1.10; 95% CI 1.06–1.14; *P* < 0.001). These associations were stronger among women with BMI < 25 kg/m² compared with those with BMI ≥ 25 kg/m² (*P* for interaction < 0.0001), and among women with waist circumference < 85 cm compared with those with waist circumference ≥ 85 cm (*P* for interaction = 0.0005). No association was observed according to antihypertensive medication class.

**Conclusions:**

Hypertension identified in early adulthood was independently and progressively associated with incident uterine fibroids in reproductive-age women. These findings suggest that blood pressure dysregulation during early adulthood may be relevant to uterine fibroid risk in young women.

## 1 Introduction

Uterine fibroids are the most common benign tumors among women and develop primarily during the reproductive years. Approximately 70% of women may develop uterine fibroids in their lifetime, and approximately 25% of reproductive-age women are treated for uterine fibroids (1). They are a major cause of gynecologic morbidity, with symptoms such as abnormal uterine bleeding, dysmenorrhea, and pelvic pain occurring in approximately 30–40% of cases (2, 3), and may also be associated with infertility, miscarriage, and adverse pregnancy outcomes (3–5). Given their increasing incidence and effects on quality of life, reproductive outcomes, and healthcare utilization, uterine fibroids impose substantial clinical and socioeconomic burdens (6–9). Although established risk factors include race, age, early menarche, parity, obesity, and genetic factors (1, 10), the full etiology of uterine fibroids remains to be determined.

Estrogen and progesterone are central regulators of fibroid growth; however, increasing evidence suggests that vascular remodeling, extracellular matrix accumulation, angiogenesis, and growth factor signaling also contribute to fibroid development (11–13). Hypertension (HTN) is increasingly recognized as a systemic condition characterized not only by elevated blood pressure (BP) but also by endocrine–vascular dysregulation, including activation of the renin–angiotensin–aldosterone system, endothelial dysfunction, and chronic inflammation (11, 14–17); these pathways overlap with biological processes implicated in uterine fibroid development. However, whether hypertension identified in early adulthood is associated with the subsequent development of uterine fibroids remains insufficiently understood.

Previous studies examining the association between BP and uterine fibroids have been limited by small sample sizes, cross-sectional designs, or reliance on self-reported BP, and few have focused specifically on young women (18). Moreover, hypertension has generally been treated as a conventional cardiovascular risk factor rather than as a potential marker of endocrine–vascular dysregulation relevant to reproductive biology (12, 18). Therefore, we aimed to investigate the association between hypertension identified in early adulthood and incident uterine fibroids in a large nationwide cohort of reproductive-age women.

## 2 Materials and methods

### 2.1 Data source and study setting

We obtained patient information from the Korean National Health Insurance Service (NHIS) claims database. As a single insurer system managed by the government, the NHIS provides mandatory comprehensive medical coverage to 97% of the Korean population, medical aid to the 3% of the population with the lowest incomes (19), free biennial cardiovascular health screenings to all Korean individuals aged ≥ 40 years and all employees regardless of age, and annual health screenings for physical laborers.

Based on an approximate participation rate of 70% (20), the NHIS maintains an extensive health information database that consists of qualification (age, sex, income, region, and type of eligibility), claims including diagnoses based on the International Classification of Diseases 10^th^ revision (ICD-10) and prescription statements, health check-up, and death information databases (21).

### 2.2 Study population

**Figure 1:**
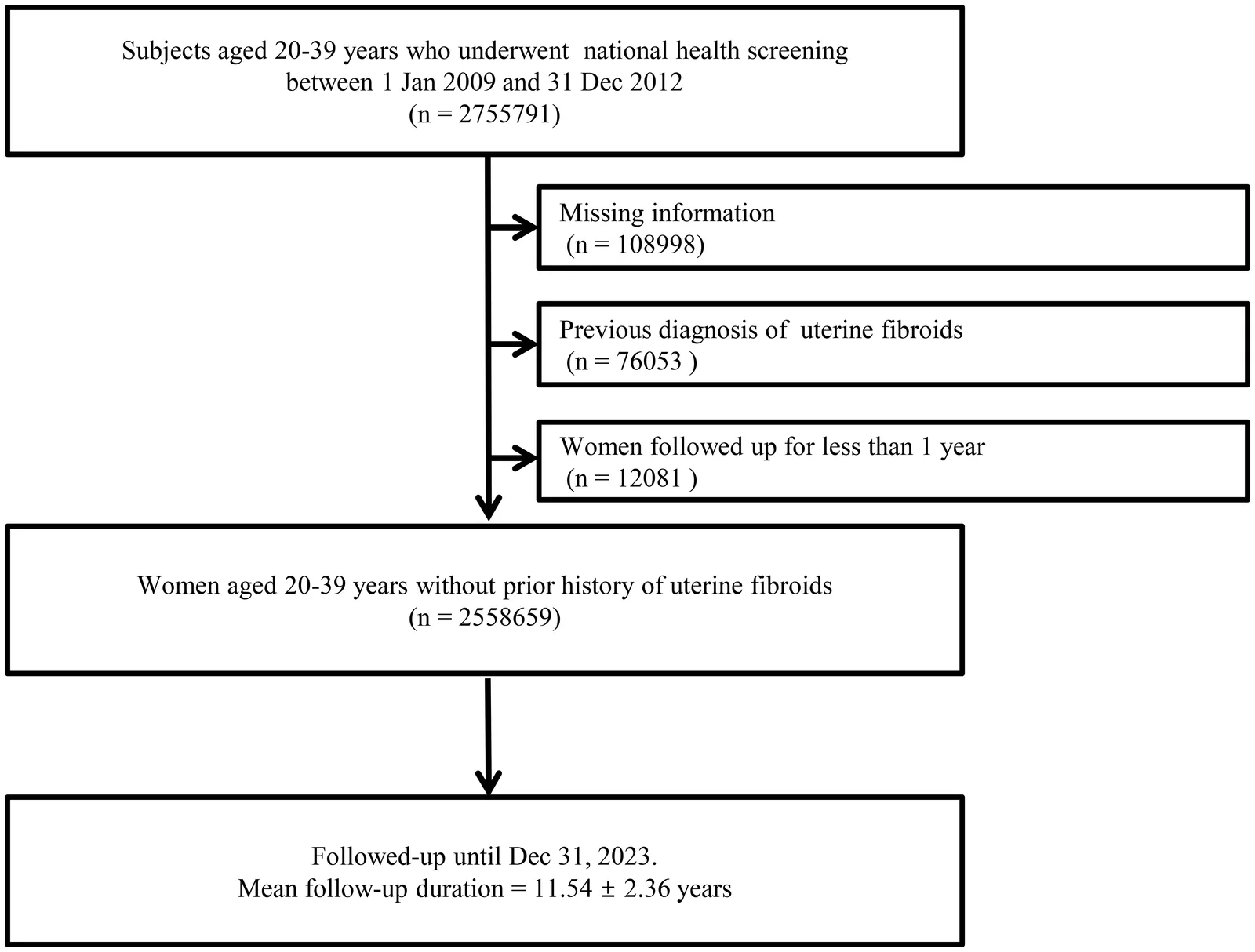
Flow chart of the study population.

Among 2,755,791 women aged 20–39 years who underwent health screenings from January 1, 2009, to December 31, 2012, 108,998 were excluded due to missing data on smoking status, alcohol consumption, regular exercise, and/or comorbidities (hypertension, diabetes, dyslipidemia). Of the remaining 2,646,793 women, 76,053 who had at least one inpatient claim or two outpatient claims with a diagnosis of leiomyoma based on the ICD-10 code D25 and 12,081 women who were followed for < 1 year were also excluded. In total, 2,558,659 eligible women aged 20–39 years with no prior history of leiomyoma were enrolled (**Figure 1**). The cohort was followed from baseline to the date of the first diagnosis of uterine fibroid, time of death, or until the end of the study period (December 31, 2023), whichever came first. The protocol for this study was approved by the Institutional Review Board of Catholic Medical Center (no. VC25ZISI0040), and the need for informed consent was waived because the data were public and anonymized based on confidentiality guidelines.

### 2.3 Exposure: BP

#### 2.3.1 BP categories

BP was measured according to the Korean Society of Hypertension guidelines (22). A trained clinician measured participants’ brachial BPs after they had been seated for 5 min with their arm in the appropriate position. The average of two brachial BP measurements was calculated. The study population was categorized into the following four subgroups based on BP measured at the baseline health screening and self-reported history of antihypertensive medication use: 1) normotensive, with systolic BP < 120 mmHg, diastolic BP < 80 mmHg, and no antihypertensive medication use; 2) elevated BP and stage 1 hypertension, with systolic BP 120−139 mmHg or diastolic BP 80−89 mmHg, and no antihypertensive medication use; 3) stage 2 hypertension, with systolic BP ≥ 140 mmHg or diastolic BP ≥ 90 mmHg, and no antihypertensive medication use; and 4) treated hypertension, in case of any antihypertensive medication history that corresponds to the answers on the health screening questionnaire (23, 24).

#### 2.3.2 Duration of hypertension

Hypertension duration was defined based on the duration of hypertension claims prior to baseline using claims data available from 2002 and categorized as < 5 years or ≥ 5 years. This variable was restricted to women with treated hypertension, as reliable longitudinal information on hypertension duration was limited to this group based on claims data.

#### 2.3.3 Antihypertensive medication

Among women with treated hypertension, medications were classified into angiotensin-converting enzyme inhibitors (ACEi), angiotensin receptor blockers (ARB), beta-blockers (BB), calcium channel blockers (CCB), and diuretics based on claims records, defined as at least one prescription with a hypertension diagnosis code within 1 year prior to baseline. Use of these medications was not mutually exclusive, as patients could receive more than one class of medication.

### 2.4 Study outcome: incident uterine fibroids

The primary outcome was incident uterine fibroids, diagnosed during the follow-up period. According to ICD-10 clinical diagnostic codes, for women who had at least two outpatient or one inpatient claim, emergency room visits for D25 after the date of their health examination were classified as having newly diagnosed uterine fibroids. Only patients whose first diagnosis occurred within the observation period were included.

Surgically treated uterine fibroids were identified among women who met the primary outcome definition during follow-up. Using procedure codes for myomectomy (R4123–R4129, R4131–R4132) and hysterectomy (R0141, R0142, R4130, R4147, R4148), cases were further categorized into two groups: (1) myomectomy only, and (2) myomectomy or hysterectomy.

### 2.5 Covariates

Body mass index (BMI) was calculated as weight (kg) divided by the square of height (m). Information on each individual’s lifestyle was categorized from the National Health Screening Program self-questionnaire. Smoking status was categorized into non-, ex-, and current smokers. Individuals who consumed ≥ 30 g of alcohol per day were classified as heavy alcohol consumers (25). Regular exercise was defined as performing > 30 min of moderate physical activity ≥ 5 times per week or > 20 min of strenuous physical activity ≥ 3 times per week (26). Comorbidities, including diabetes, hypertension, and dyslipidemia, were based on ICD-10 codes from each patient’s past medical history (27). The estimated glomerular filtration rate (eGFR) was calculated using the Chronic Kidney Disease Epidemiology Collaboration equations. An eGFR < 60 mL/min/1.73 m^2^ was considered indicative of chronic kidney disease. Blood samples for the measurement of serum fasting glucose and lipid levels were drawn after an overnight fast.

Women were classified as having diabetes mellitus if: 1) they had at least one claim with a diagnosis of diabetes based on the ICD-10 code E11-14 and prescribed at least one antidiabetic drug at any time over a year, to exclude prediabetic or nondiabetic individuals, or 2) they had a fasting plasma glucose ≥ 126 mg/dL during a health examination (newly diagnosed diabetes) (21). Women were classified as having hypertension if: 1) they had at least one claim with a diagnosis of hypertension based on ICD-10 code I10−13 or I15, and were prescribed at least one antihypertensive agent at any time over a year, to exclude individuals with elevated BP or stage 1 hypertension (28), which was classified as prehypertension prior to the publication of the 2017 American College of Cardiology/American Heart Association high Blood Pressure clinical practice guidelines, or 2) systolic BP/diastolic BP ≥ 140/90 mmHg. The hospitals where each of these health examinations was performed were certified by the NHIS and subjected to regular quality control (19).

Healthcare utilization was defined as the average annual number of outpatient visits during the follow-up period.

### 2.6 Statistical analysis

Continuous variables were presented as mean ± standard deviation (SD), and categorical variables as numbers and percentages. Incidence rates of uterine fibroids were expressed as the number of new diagnoses per 1,000 person-years. We used the Cox proportional hazards regression analysis to calculate hazard ratios (HRs) and 95% confidence intervals (CIs) for the associations between various factors and the incidence of uterine fibroids. The proportional hazards assumption was assessed using Schoenfeld residuals and log-log plots, and no violations were observed.

Potential confounders or effect modifiers were identified *a priori* based on a literature review and presumed causal relationships among the covariates. Model 1 was unadjusted, whereas Model 2 was adjusted for age, diabetes, dyslipidemia, chronic kidney disease, alcohol consumption, smoking, regular exercise, BMI, and income. Model 3 was further adjusted for the average annual number of outpatient visits in addition to the covariates included in Model 2.

Additional analyses restricted to women with treated hypertension were conducted to examine the association according to hypertension duration and antihypertensive medication class. For medication class analyses, each class was evaluated to assess whether the association between treated hypertension and uterine fibroid risk differed by medication type. Sensitivity analyses were conducted by restricting the primary outcome to surgically treated cases to evaluate the associations when limited to clinically significant uterine fibroids.

To facilitate clinical interpretation, we estimated 5-year absolute risks, absolute risk differences, and number needed to expose (NNE) from multivariable-adjusted models. NNE was defined as the number of exposed women in a given BP category associated with one additional case of uterine fibroids over 5 years compared with the normotensive group, and was calculated as the inverse of the absolute risk difference. The population attributable fraction (PAF) was estimated using the prevalence of elevated BP and hypertension at baseline and the corresponding hazard ratios.

All statistical analyses were performed using SAS version 9.4 (SAS Institute Inc., Cary, NC, USA).

### 2.7 Core outcome sets and patient involvement

The primary outcome of this study was the incidence of uterine fibroids. While there is currently no universally established core outcome set for uterine fibroid research, we selected outcomes that are clinically relevant and widely reported in prior studies, including new diagnosis based on ICD-10 codes. Patient involvement in the design or conduct of this study was not directly implemented, as the study used a retrospective analysis of anonymized claims data from the Korean NHIS.

## 3 Results

### 3.1 Baseline characteristics

During the mean follow-up period of 11.54 ± 2.36 years, 373,379 (14.6%) of 2,558,659 initially eligible women had newly identified uterine fibroids. Patients with uterine fibroids were older than those without (31.19 ± 5.10 vs. 29.39 ± 5.17 years, respectively), had more comorbidities such as diabetes (0.99 vs. 0.93%), hypertension (3.06 vs. 2.36%), and dyslipidemia (3.80 vs. 3.69%), were less likely to have a history of smoking (never smoker, 90.42 vs. 90.52%; ex-smoker, 3.64 vs. 3.56%; current smoker, 5.93 vs. 5.91%), were less likely to be heavy drinkers (4.94 vs. 5.03%), participated in more regular physical activities (9.83 vs. 9.50%), were more likely to have higher BMIs (21.54 ± 3.20 vs. 21.31± 3.26), were less likely to have lower income (28.28 vs. 28.99%), had a higher systolic BP (111.84 ± 11.65 vs. 111.11 ± 11.49 mmHg), higher diastolic BP (70.27 ± 8.62 vs. 69.76 ± 8.49 mmHg), higher total cholesterol (178.9 ± 30.49 mg/dL), higher low density lipoprotein (100.35 ± 30.77 vs. 98.94 ± 30.86 mg/dL), and lower high density lipoprotein (62.76 ± 26.42 vs. 63.49 ± 27.67 mg/dL) (**Table 1**).

**Table 1:** Baseline characteristics of study subjects (n=2,558,659).

Characteristic	Total participants	Uterine fibroids incidence		P value
		No	Yes	
N	2558659	2185280 (85.4)	373379 (14.6)	
Age, years	29.65 ± 5.2	29.39 ± 5.17	31.19 ± 5.1	< 0.0001
20-29	1344212 (52.54)	1195477 (54.71)	148735 (39.83)	
30-39	1214447 (47.46)	989803 (45.29)	224644 (60.17)	
Duration of follow up, years	11.54 ± 2.36	12.22 ± 1.19	7.55 ± 3.37	< 0.0001
Comorbidities				
Diabetes	23944 (0.94)	20247 (0.93)	3697 (0.99)	0.0002
Hypertension	62972 (2.46)	51565 (2.36)	11407 (3.06)	< 0.0001
Dyslipidemia	94786(3.70)	80612 (3.69)	14174 (3.80)	0.0013
Chronic kidney disease	52772 (2.06)	45164 (2.07)	7608 (2.04)	0.2471
Smoking status				0.0415
Never	2315807 (90.51)	1978189 (90.52)	337618 (90.42)	
Ex-smoker	91486 (3.58)	77879 (3.56)	13607 (3.64)	
Current smoker	151366 (5.92)	129212 (5.91)	22154 (5.93)	
Alcohol consumption				< 0.0001
None	1391919 (54.40)	1189729 (54.44)	202190 (54.15)	
Mild	1038399 (40.58)	885673 (40.53)	152726 (40.90)	
Heavy	128341 (5.02)	109878 (5.03)	18463 (4.94)	
Regular exercise (yes)	244241 (9.55)	207542 (9.50)	36699 (9.83)	< 0.0001
Body mass index, mean, kg/m2	21.34 ± 3.25	21.31 ± 3.26	21.54 ± 3.20	< 0.0001
< 18.5	382538 (14.95)	334768 (15.32)	47770 (12.79)	< 0.0001
18.5 to < 23	1580005 (61.75)	1349418 (61.75)	230587 (61.76)	< 0.0001
23 to < 25	295689 (11.56)	248289 (11.36)	47400 (12.69)	< 0.0001
25 to <30	240418 (9.40)	201360 (9.21)	39058 (10.46)	< 0.0001
≥ 30	60009 (2.35)	51445 (2.35)	8564 (2.29)	< 0.0001
Waist Circumference ≥ 85 cm	156578 (6.12)	132872 (6.08)	23706 (6.35)	< 0.0001
Low income (%)	739145 (28.89)	633552 (28.99)	105593 (28.28)	< 0.0001
Blood pressure (BP), mean, mmHg				
Systolic	111.22 ± 11.52	111.11 ± 11.49	111.84 ± 11.65	< 0.0001
Diastolic	69.83 ± 8.51	69.76 ± 8.49	70.27 ± 8.62	< 0.0001
High density lipoprotein, mg/dl	63.26 ± 23.54	63.36 ± 23.50	62.70 ± 23.81	< 0.0001
Low Density Lipoprotein, mg/dl	99.15 ± 30.85	98.94 ± 30.86	100.35 ± 30.77	< 0.0001
Triglyceride*, mg/dl	71.79 (71.74–71.83)	71.5 (71.46–71.55)	73.47 (73.36–73.58)	< 0.0001
Average annual outpatient visits, n	11.27 ± 7.67	11.08 ± 7.53	12.36 ± 8.35	< 0.0001

Data are expressed as the mean ± standard deviation, SD, or n (%).

^*^ Geometric mean (95% C.I).

### 3.2 Risk of uterine fibroids varies based on BP categorization

**Figure 2:**
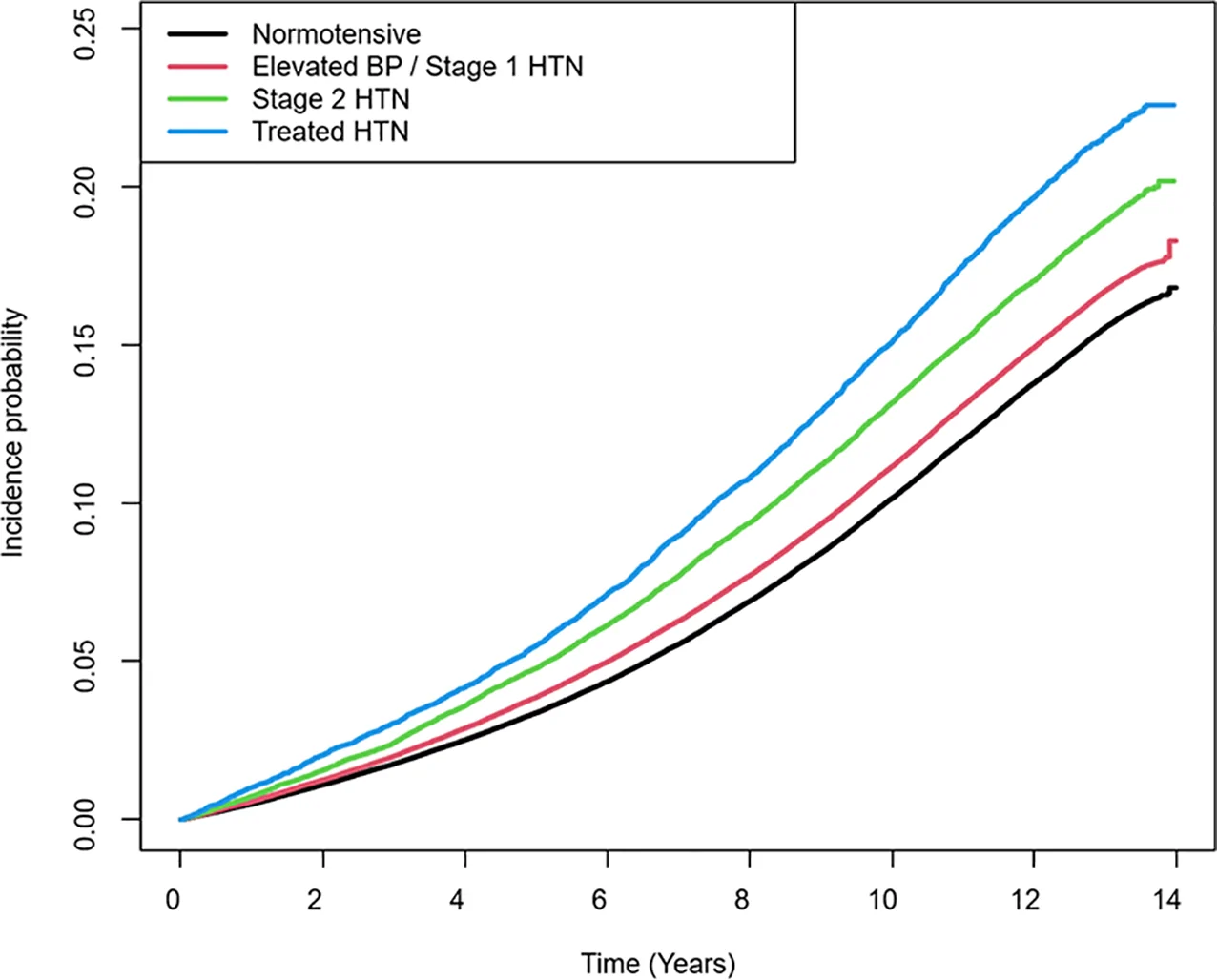
Cumulative incidence of uterine fibroids according to blood pressure (BP) categories. The cumulative incidence of uterine fibroids according to BP categories was calculated using Kaplan–Meier curves, and the log-rank test was performed to analyze differences amongst the groups.

A graded association was observed across blood pressure categories. Stage 2 hypertension (systolic BP ≥ 140 mmHg or diastolic BP ≥ 90 mmHg) without treatment was associated with a higher risk of uterine fibroids (adjusted HR: 1.06; 95% CI: 1.04–1.09; P < 0.001) compared to stage 1 hypertension and elevated BP (adjusted HR: 1.04; 95% CI: 1.03–1.05; P < 0.001) or a normotensive state (reference) after adjusting for age, alcohol consumption, smoking, regular exercise, income, BMI, diabetes, dyslipidemia, chronic kidney disease, and healthcare utilization (**Table 2**). Among the BP categories, the risk of uterine fibroids was highest in women with treated hypertension (adjusted HR: 1.10; 95% CI: 1.06–1.14; P < 0.001) (**Table 2**). This graded association between each stage of BP and the risk of uterine fibroids was evident in women with BMI < 25 kg/m2 rather than in women with BMI ≥ 25 kg/m2 (P for interaction < 0.0001), and waist circumference (WC) < 85 cm (P for interaction = 0.0005). No significant difference was found between women aged 20–29 and 30–39 years (**Table 3**). The cumulative incidence of uterine fibroids for up to 14 years according to the BP categories is shown using Kaplan–Meier curves (**Figure 2**). The cumulative incidence of uterine fibroids increased with increasing hypertension stages, and was highest in women with treated hypertension (all log-rank P < 0.0001). In additional analyses restricted to women with treated hypertension, longer hypertension duration was associated with a higher risk of uterine fibroids. Compared with women with a hypertension duration of < 5 years, those with a duration of ≥ 5 years exhibited an increased risk across all models (**Table 4**). No specific antihypertensive medication class was independently associated with uterine fibroid risk after adjustment for potential confounders (**Table 5**).

**Table 2:** Hazard ratios and 95% confidence intervals of uterine fibroids by BP.

	Subjects	Uterine fibroids	Follow-up duration	Incidence rate	HR (95% confidence intervals)		
					Model 1	Model 2	Model 3
BP, 4 levels							
Normotensive (< 120/80)	1754064	253042	20636617	12.26	1 (Reference)	1 (Reference)	1 (Reference)
Elevated BP and stage 1 hypertension (< 140/90)	710623	108930	8173564	13.33	1.09* (1.08, 1.10)	1.04* (1.03, 1.04)	1.04* (1.03, 1.05)
Stage 2 hypertension (≥ 140/90)	45723	7953	516424	15.40	1.27* (1.24, 1.29)	1.07* (1.04, 1.09)	1.06* (1.04, 1.09)
Treated hypertension	17249	3454	192950	17.90	1.47* (1.43, 1.53)	1.16* (1.12, 1.20)	1.10* (1.06, 1.14)

Subjects, n.; Uterine fibroids, n; Follow-up duration, person-years; Incidence rate, per 1000 person-years.

Model 1: unadjusted.

Model 2: adjusted for age, alcohol consumption, smoking, regular exercise, income, body mass index, dyslipidemia, diabetes, chronic kidney disease.

Model 3: further adjusted for the average annual number of outpatient visits in addition to the covariates included in Model 2.

^*^ *P* < 0.0001 compared with reference values.

**Table 3:** Subgroup analysis for hazard ratios of uterine fibroids by BP categories for subgroups.

Subgroup	BP, 4 levels	Incidence rate	HR ^a^ (95% CI)	P for interaction
Age				
20-29^b^	Normotensive	9.31	1 (Ref.)	0.2118
	Elevated BP and stage 1 hypertension	9.70	1.029 (1.018–1.041)	
	Stage 2 hypertension	10.34	1.066 (1.019–1.116)	
	Treated hypertension	11.63	1.225 (1.124–1.336)	
30-39^c^	Normotensive	15.91	1(Ref.)	
	Elevated BP and stage 1 hypertension	17.05	1.042 (1.033–1.052)	
	Stage 2 hypertension	18.26	1.072 (1.044–1.100)	
	Treated hypertension	19.80	1.154 (1.113–1.198)	
BMI				
< 25^d^	Normotensive	12.16	1 (Ref.)	< 0.0001
	Elevated BP and stage 1 hypertension	13.15	1.039 (1.030–1.047)	
	Stage 2 hypertension	15.87	1.115 (1.083–1.148)	
	Treated hypertension	17.71	1.188 (1.139–1.239)	
≥25^e^	Normotensive	13.48	1 (Ref.)	
	Elevated BP and stage 1 hypertension	14.09	1.025 (1.005–1.044)	
	Stage 2 hypertension	14.78	1.001 (0.965–1.039)	
	Treated hypertension	18.26	1.105 (1.043–1.171)	
WC				
< 85^f^	Normotensive	12.24	1 (Ref.)	0.0005
	Elevated BP and stage 1 hypertension	13.30	1.037 (1.029–1.045)	
	Stage 2 hypertension	16.04	1.107 (1.078–1.136)	
	Treated hypertension	17.94	1.169 (1.125–1.214)	
≥85^g^	Normotensive	12.71	1 (Ref.)	
	Elevated BP and stage 1 hypertension	13.53	1.033 (1.005–1.061)	
	Stage 2 hypertension	13.80	0.987 (0.941–1.036)	
	Treated hypertension	17.77	1.140 (1.058–1.228)	

BP, Blood pressure; Subjects, n; Uterine fibroids, n; Follow-up duration, person-years; Incidence rate, per 1,000 person-years; BMI, Body Mass Index, kg/m^2^; WC, Waist Circumference, centimeter;.

^a^adjusted for age, alcohol consumption, smoking, regular exercise, income, BMI, dyslipidemia, chronic kidney disease, diabetes; ^b^*P* for trend < 0.0001; ^c^*P* for trend < 0.0001; ^d^*P* for trend < 0.0001; ^e^*P* for trend = 0.0062; ^f^*P* for trend < 0.0001; ^g^*P* for trend = 0.0279.

**Table 4:** Hazard ratios and 95% confidence intervals of uterine fibroids according to blood pressure and hypertension duration.

BP	Subjects	Uterine fibroids	Follow-up duration	Incidence rate	HR (95% confidence intervals)		
					Model 1	Model 2	Model 3
Normotensive	1785064	253042	20636617	12.26	1 (Reference)	1 (Reference)	1 (Reference)
Elevated BP and stage 1 hypertension	710623	108930	8173564	13.33	1.089* (1.081, 1.096)	1.037* (1.030, 1.045)	1.039* (1.031, 1.046)
Stage 2 hypertension	45723	7953	516424	15.40	1.263* (1.235, 1.291)	1.066* (1.042, 1.091)	1.062* (1.038, 1.086)
Treated hypertension < 5 years	13629	2690	153395	17.60	1.444* (1.391, 1.500)	1.152* (1.109, 1.196)	1.094* (1.053, 1.136)
Treated hypertension ≥ 5 years	3620	764	39555	19.37	1.618* (1.508, 1.736)	1.210* (1.128, 1.299)	1.115* (1.039, 1.197)

Subjects, n.; Uterine fibroids, n; Follow-up duration, person-years; Incidence rate, per 1000 person-years.

Model 1: unadjusted.

Model 2: adjusted for age, alcohol consumption, smoking, regular exercise, income, body mass index, dyslipidemia, diabetes, chronic kidney disease.

Model 3: further adjusted for the average annual number of outpatient visits in addition to the covariates included in Model 2.

^*^ *P* < 0.0001 compared with reference values.

**Table 5:** Association between antihypertensive medication classes and risk of uterine fibroids among women with treated hypertension.

	Subjects	Events	Incidence rate	HR (95% confidence interval)		
				Model 1	Model 2	Model 3
ACEi						
No	16179	3252	17.98	1 (Reference)	1 (Reference)	1 (Reference)
Yes	1070	202	16.77	0.932 (0.809, 1.074)	0.991 (0.859, 1.143)	0.984 (0.853, 1.134)
ARB						
No	9467	1825	17.06	1 (Reference)	1 (Reference)	1 (Reference)
Yes	7782	1629	18.95	1.122 (1.050, 1.200)	1.060 (0.990, 1.136)	1.053 (0.983, 1.128)
Beta blocker						
No	12937	2584	17.89	1 (Reference)	1 (Reference)	1 (Reference)
Yes	4312	870	17.95	1.003 (0.929, 1.083)	1.009 (0.935, 1.090)	1.009 (0.935, 1.090)
Diuretics						
No	14237	2805	17.55	1 (Reference)	1 (Reference)	1 (Reference)
Yes	3012	649	19.57	1.122 (1.030, 1.222)	1.035 (0.948, 1.130)	1.032 (0.946, 1.127)
CCB						
No	12203	2395	17.43	1 (Reference)	1 (Reference)	1 (Reference)
Yes	5046	1059	19.06	1.102 (1.025, 1.185)	1.010 (0.938, 1.088)	1.006 (0.934, 1.084)

Subjects, n.; Events, n; Incidence rate, per 1000 person-years.

ACEi, angiotensin-converting enzyme inhibitors; ARB, angiotensin receptor blockers; BB, beta-blockers; CCB, calcium channel blockers.

Model 1: unadjusted.

Model 2: adjusted for age, alcohol consumption, smoking, regular exercise, income, body mass index, dyslipidemia, diabetes, chronic kidney disease.

Model 3: further adjusted for the average annual number of outpatient visits in addition to the covariates included in Model 2.

### 3.3 Sensitivity analyses for surgically treated uterine fibroids

#### 3.3.1 Myomectomy

Increased risk of uterine fibroids treated with myomectomy was shown in women with elevated blood pressure and stage 1 hypertension (adjusted HR: 1.053; 95% CI: 1.035–1.071) and stage 2 hypertension (adjusted HR: 1.076; 95% CI: 1.019–1.137), whereas no significant difference was shown in women with treated hypertension (adjusted HR: 0.920; 95% CI: 0.838–1.010) compared to normotensive women (**Table 6**).

**Table 6:** Sensitivity analysis of the association between blood pressure categories and surgically treated uterine fibroids.

BP	Subjects	Events	Incidence rate	HR (95% confidence intervals)		
				Model 1	Model 2	Model 3
Myomectomy						
Normotensive	1785064	46200	1.97	1 (Reference)	1 (Reference)	1 (Reference)
Elevated BP and stage 1 hypertension	710623	19825	2.13	1.078* (1.061, 1.096)	1.052* (1.035, 1.070)	1.053* (1.035, 1.071)
Stage 2 hypertension	45723	1366	2.29	1.166* (1.105, 1.231)	1.078* (1.020, 1.138)	1.076* (1.019, 1.137)
Treated hypertension	17249	456	2.01	1.018 (0.928, 1.117)	0.941 (0.858, 1.033)	0.920 (0.838, 1.010)
Myomectomy or Hysterectomy						
Normotensive	1785064	64611	2.76	1 (Reference)	1 (Reference)	1 (Reference)
Elevated BP and stage 1 hypertension	710623	30209	3.25	1.178* (1.162, 1.194)	1.078* (1.063, 1.093)	1.078* (1.063, 1.094)
Stage 2 hypertension	45723	2409	4.06	1.480* (1.421, 1.541)	1.113* (1.067, 1.160)	1.110* (1.065, 1.157)
Treated hypertension	17249	980	4.36	1.580* (1.483, 1.683)	1.122* (1.053, 1.196)	1.073* (1.006, 1.144)

Subjects, n.; Events, n; Incidence rate, per 1000 person-years.

Model 1: unadjusted.

Model 2: adjusted for age, alcohol consumption, smoking, regular exercise, income, body mass index, dyslipidemia, diabetes, chronic kidney disease.

Model 3: further adjusted for the average annual number of outpatient visits in addition to the covariates included in Model 2.

^*^ *P* < 0.0001 compared with reference values.

#### 3.3.2 Myomectomy or hysterectomy

All three BP categories were associated with an increased risk of uterine fibroids treated with myomectomy or hysterectomy compared to normotensive group (adjusted HR: 1.078; 95% CI: 1.063–1.094, adjusted HR: 1.110; 95% CI: 1.065–1.157, adjusted HR: 1.073; 95% CI: 1.006–1.144, respectively). The risk in women with treated hypertension was lower than that in women with stage 2 hypertension (**Table 6**).

### 3.4 Absolute and attributable risk associated with hypertension and uterine fibroids

The 5-year absolute risk of uterine fibroids increased from 3.34% in normotensive individuals to 3.47% in women with elevated BP and stage 1 hypertension, 3.55% in women with stage 2 hypertension, and 3.67% in those with treated hypertension. These correspond to absolute risk differences of 0.13–0.32 percentage points and NNE values ranging from 311 to 788 (**Supplementary Table 1**). The population attributable fraction for elevated BP and hypertension was 1.24%.

## 4 Discussion

In this nationwide population-based cohort study of women aged 20–39 years, higher BP categories were independently and progressively associated with an increased risk of incident uterine fibroids after adjustment for potential confounders, including healthcare utilization. The association was more prominent among women with lower BMI or waist circumference. Women with treated hypertension also showed higher fibroid risk, particularly when hypertension duration was ≥ 5 years. The absence of an independent association according to antihypertensive medication class suggests that this finding may reflect greater hypertension burden, including longer duration, greater severity, or suboptimal BP control, rather than medication-specific effects.

Previous reports have suggested an association between hypertension and uterine fibroids, although the causal relationship between them remains unclear (17, 29, 30). A cross-sectional study of women in Finland showed an increase in uterine fibroids among women with hypertension compared to those with normotension (17). One prospective study showed a positive association between elevated BP and the risk for clinically detected uterine fibroids; however, BP was self-reported, and the analysis focused mainly on diastolic BP (16). Hypertension was also suggested as a risk factor for uterine fibroids in a small retrospective case-control study among women who underwent surgical treatment for uterine fibroids (31). The graded association between BP levels and uterine fibroid risk in this observational cohort of young women may be consistent with shared endocrine–vascular processes relevant to fibroid development, although causality cannot be determined.

Hypertension, characterized by chronic hemodynamic stress and endothelial dysfunction, is a proatherogenic state that may promote smooth muscle cell injury and maladaptive repair processes in a manner analogous to atherosclerotic changes in arterial smooth muscle (30). In vascular tissue, these processes lead to increased permeability, smooth muscle cell migration and proliferation, extracellular matrix deposition, and the formation of fibrous plaque or fibroid. The process of uterine smooth muscle cell injury is closely similar to the histopathologic characteristics of those mentioned above, which trigger the monoclonal expansion of uterine smooth muscle cells from the uterine arteries, myometrium, or connective tissue, resulting in the formation of uterine fibroids (32).

Activation of the renin–angiotensin–aldosterone system represents a key endocrine pathway linking hypertension to fibroid development. In hypertension, sustained activation of the renin–angiotensin–aldosterone system leads to elevated levels of angiotensin II, which mediates many of the hemodynamic and tissue-remodeling effects associated with chronically increased BP (33). Angiotensin II stimulates transforming growth factor-β signaling that enhances extracellular matrix production and reduces its degradation, leading to the accumulation of extracellular matrix, one of the components which distinguish uterine fibroids (34–36). In addition, angiotensin II exerts mitogenic effects on smooth muscle cells through activation of transforming growth factor β signaling, further facilitating fibroid growth (6, 36, 37). These mechanisms may be particularly relevant in hypertension identified in early adulthood, where prolonged exposure to endocrine and inflammatory stimuli occurs during hormonally active reproductive years (38, 39).

The stronger association among women with lower BMI may suggest that hypertension-related endocrine–vascular pathways are not fully explained by obesity-related estrogenic mechanisms. Obesity-related estrogen exposure and metabolic inflammation may be dominant contributors to fibroid risk in women with higher BMI, whereas hypertension-related vascular remodeling, endothelial dysfunction, renin–angiotensin–aldosterone system activation, and low-grade inflammation may be more discernible in women with lower BMI. This interpretation remains speculative and should be examined in future studies with detailed hormonal and metabolic data.

Individuals with hypertension identified in early adulthood are more likely to have an inherited risk trait and a higher long-term risk for cardiovascular disease and mortality than those with hypertension identified later in life (38, 40–42). These findings suggest that BP dysregulation during early adulthood may reflect sustained vascular, inflammatory, and endocrine alterations during the reproductive years. In this study of young women aged 20–39 years with hypertension identified in early adulthood, the higher fibroid risk observed among women with treated hypertension may reflect greater hypertension burden rather than a direct pharmacologic effect, as this association was more prominent among women with longer hypertension duration and no medication class-specific association was observed. Prior evidence that awareness, treatment, and control of hypertension are substantially lower among younger individuals than among older adults (43–45) supports these findings, suggesting that treatment status in young women may not necessarily indicate adequate blood pressure control. The difference between our findings and those of a previous midlife cohort study (18) may therefore be partly explained by differences in age, hypertension exposure, treatment initiation, and BP control.

In sensitivity analyses of surgically treated uterine fibroids, women with treated hypertension showed a lower risk than those with stage 2 hypertension. This finding may suggest that current elevated BP status, rather than treatment status alone, may be relevant to clinically significant fibroid outcomes. However, this result should be interpreted with caution because surgically treated outcomes may be influenced by treatment-seeking behavior, surgical decision-making, and disease severity.

Nevertheless, to the best of our knowledge, this study is the first to evaluate the association between hypertension status and incident fibroids in young reproductive-age women using a large nationwide cohort. Although the individual-level effect sizes were modest, the observed associations may be relevant at the population level given the high prevalence of both elevated BP and uterine fibroids. By identifying hypertension status as a previously underrecognized factor associated with uterine fibroid risk, these findings may help improve risk awareness and guide future prevention-oriented research.

### 4.1 Limitations

This study has some limitations. First, the incidence of uterine fibroids was determined by claims data alone. Therefore, it is possible that not all cases of fibroids were identified, and detailed clinical information on size, number, or location of uterine fibroids could not be obtained. The most common and easily accessible diagnostic tool for detecting uterine fibroids is pelvic sonography, which is not part of the biennial national health insurance examination, and not all uterine fibroids are symptomatic or clinically detected. Under these conditions, cases in which women had uterine fibroids without any related symptoms and were not screened with pelvic sonography might not have been included. Fibroid cases can also be missed if the physician did not add the D25 code due to the absence of an insurance claim. On the other hand, frequent healthcare utilization by women with hypertension may contribute to higher likelihood of incidental fibroid detection due to increased surveillance. To address this potential surveillance bias between women with normotension and those with hypertension, we adjusted for healthcare utilization by including the average annual number of outpatient visits as a covariate. In addition, as the endpoints of this study were defined based on the claims data, misclassification is possible, although this approach has been widely used in previous studies. In the sensitivity analysis of surgically treated uterine fibroids, hysterectomy was identified when a hysterectomy procedure code was accompanied by a uterine fibroid diagnosis. However, we could not fully exclude hysterectomies performed primarily for indications other than fibroids when other gynecologic conditions coexisted with a fibroid diagnosis. Therefore, the results of this analysis should be interpreted cautiously. Second, reproductive and gynecologic history was not obtained as the screening was performed using a general health examination which did not provide gynecologic history. Therefore, women with reproductive characteristics associated with a lower risk of uterine fibroids, such as parity, prior delivery, and lactation, as well as other factors that have been reported to be associated with fibroid risk, including age at menarche, menstruation characteristics, age at first birth, hormonal contraceptive use, and family history of uterine fibroids, may have been included in the study population, potentially contributing to residual confounding, the direction and magnitude of which could not be determined. Some of these variables may also be related to hypertension status. In addition, women who had undergone hysterectomy might have been included in the study population, which may have affected the result of the study, even though hysterectomy is rare in women < 40 years of age. Third, hypertension status was defined based on BP measured at the baseline health screening and history of antihypertensive medication use; therefore, the age at hypertension onset, incident hypertension or changes in BP status during follow-up, BP control over time, and medication adherence could not be assessed, and detailed treatment regimens among treated women were not included in the analysis. These factors may have influenced the observed associations and may partly reflect differences in hypertension burden, severity, or control. Shared endocrine–vascular pathways may partly underlie the observed associations, although the possibility of reverse causality cannot be entirely excluded. Fourth, the magnitude of the effect sizes was relatively small although statistically significant associations were observed. Therefore, the clinical significance at the individual level may be limited. Finally, this study showed only the results of individuals of Asian ethnicity, limiting the generalizability of our results.

## 5 Conclusion

In this nationwide cohort of reproductive-age women, hypertension identified in early adulthood was associated with a modest but graded increase in the risk of incident uterine fibroids. The small individual-level effect size indicates that these findings should not be interpreted as supporting changes in fibroid screening or clinical management based on hypertension status alone. However, given the high prevalence of both elevated BP and uterine fibroids, the observed association may have relevance for population-level risk awareness. Further studies with longitudinal BP measurements, detailed reproductive and gynecologic information, and clinically characterized fibroid outcomes are needed to clarify the biological pathways linking BP dysregulation and fibroid development and to determine whether BP status may have a role in future risk stratification or preventive strategies.

## Data Availability

The data analyzed in this study is subject to the following licenses/restrictions: Access to the dataset is restricted by the data provider due to confidentiality and privacy regulations. The data are not publicly available. Requests to access the dataset should be directed to the official data provider, the National Health Insurance Service (NHIS), through its data access application procedures.

## Supplementary material

Supplementary Table 1 Absolute and attributable risk associated with hypertension and incident uterine fibroids. NNE, number needed to expose; PAF, population attributable fraction. Absolute risks and risk differences were derived from multivariable-adjusted models. Adjusted for age, alcohol consumption, smoking, regular exercise, income, body mass index, dyslipidemia, diabetes, chronic kidney disease, and the average annual number of outpatient visits. Prevalence represents the proportion of participants with elevated blood pressure or higher at baseline in the study population. PAF was estimated for the composite exposure category of elevated blood pressure and HTN.
