# Supplementary material for: Hypertension identified in early adulthood as an endocrine–vascular risk factor for uterine fibroids in reproductive-age women: a nationwide cohort study
Source: Front Endocrinol (Lausanne). 2026 Aug 25;17:1822452. doi: 10.3389/fendo.2026.1822452 (PMC13549899; doi:10.3389/fendo.2026.1822452)
Supplement: Supplementary file 1 [file Table1.docx]

**Supplementary Table 1. Absolute and attributable risk associated with hypertension and incident uterine fibroids**

| **BP categories** | | **HR (95% CI)** | **5 year absolute risk (%)** | **Absolute risk difference (%)** | **NNE (n)** | **PAF (%)** | **Prevalence (%)** |
| --- | --- | --- | --- | --- | --- | --- | --- |
| Normotensive | 1  (Reference) | | 3.34  (3.32, 3.36) | 0  (Reference) | - | - | - |
| Elevated BP and stage 1 hypertension | 1.04  (1.03, 1.05) | | 3.47  (3.44, 3.50) | 0.13  (0.10, 0.15) | 788 |  |  |
| Stage 2 hypertension | 1.06  (1.04, 1.09) | | 3.55  (3.46, 3.63) | 0.20  (0.12, 0.28) | 491 |  |  |
| Treated hypertension | 1.10  (1.06, 1.14) | | 3.67  (3.54, 3.79) | 0.32  (0.20, 0.44) | 311 |  |  |
| Elevated BP and Hypertension | - | | - | - | - | 1.24 | 30.23 |

NNE, number needed to expose; PAF, population attributable fraction.

Absolute risks and risk differences were derived from multivariable-adjusted models.

Adjusted for age, alcohol consumption, smoking, regular exercise, income, body mass index, dyslipidemia, diabetes, chronic kidney disease, and the average annual number of outpatient visits.

Prevalence represents the proportion of participants with elevated blood pressure or higher at baseline in the study population.

PAF was estimated for the composite exposure category of elevated blood pressure and HTN.
